# Ovarian Hormones Regulate Nicotine Consumption and Accumbens Glutamatergic Plasticity in Female Rats

**DOI:** 10.1523/ENEURO.0286-21.2022

**Published:** 2022-06-23

**Authors:** Erin E. Maher, Zachary A. Kipp, Jonna M. Leyrer-Jackson, Shailesh Khatri, Emma Bondy, Genesee J. Martinez, Joshua S. Beckmann, Terry D. Hinds, Heather A. Bimonte-Nelson, Cassandra D. Gipson

**Affiliations:** 1 Department of Pharmacology and Nutritional Sciences, University of Kentucky, Lexington, KY, 40536; 2 Department of Psychology, Arizona State University, Tempe, AZ, 85287; 3 Department of Psychology, University of Kentucky, Lexington, KY, 40506; 4 Barnstable Brown Diabetes Center, University of Kentucky College of Medicine, Lexington, KY, 40536; 5 Markey Cancer Center, University of Kentucky, Lexington, KY, 40536; 6 Arizona Alzheimer’s Consortium, Phoenix, AZ 85014

**Keywords:** accumbens, estrogen, glutamate, hormones, nicotine, plasticity

## Abstract

Women report greater cigarette cravings during the menstrual cycle phase with higher circulating levels of 17β-estradiol (E2), which is metabolized to estrone (E1). Both E2 and E1 bind to estrogen receptors (ERs), which have been highly studied in the breast, uterus, and ovary. Recent studies have found that ERs are also located on GABAergic medium spiny neurons (MSNs) within the nucleus accumbens core (NAcore). Glutamatergic plasticity in NAcore MSNs is altered following nicotine use; however, it is unknown whether estrogens impact this neurobiological consequence. To test the effect of estrogen on nicotine use, we ovariectomized (OVX) female rats that then underwent nicotine self-administration acquisition and compared them to ovary-intact (sham) rats. The OVX animals then received either sesame oil (vehicle), E2, or E1+E2 supplementation for 4 or 20 d before nicotine sessions. While both ovary-intact and OVX females readily discriminated levers, OVX females consumed less nicotine than sham females. Further, neither E2 nor E1+E2 increased nicotine consumption back to sham levels following OVX, regardless of the duration of the treatment. OVX also rendered NAcore MSNs in a potentiated state following nicotine self-administration, which was reversed by 4 d of systemic E2 treatment. Finally, we found that E2 and E1+E2 increased ERα mRNA in the NAcore, but nicotine suppressed this regardless of hormone treatment. Together, these results show that estrogens regulate nicotine neurobiology, but additional factors may be required to restore nicotine consumption to ovary-intact levels.

## Significance Statement

We report that both ovariectomized (OVX) and ovary-intact female rats acquire nicotine self-administration, with ovary-intact females consuming more nicotine than OVX females. Neither treatment with 17β-estradiol (E2) or a combination of E2 and its metabolite, estrone (E1), reversed OVX-induced suppression of nicotine consumption. We further report that cessation of ovarian hormones because of OVX increased AMPA/NMDA ratios of accumbens medium spiny neurons (MSNs) in nicotine-experienced females, which is reversed by E2 supplementation. Collectively, these studies demonstrate the important contributions of estrogens to nicotine neurobiology and highlight differences in how ovarian hormones impact nicotine neurobiology and behavior. These studies may provide insight into the critical role of the hypothalamic-pituitary-ovarian axis in regulating nicotine consumption in women.

## Introduction

The menstrual cycle phase in women alters nicotine craving and propensity for relapse following abstinence (Carpenter et al., 2006; Allen et al., 2008; Franklin et al., 2008). Given that estrogen levels fluctuate throughout the menstrual cycle and higher estrogen levels are associated with increased craving and relapse vulnerability (Carpenter et al., 2006; Allen et al., 2008; Franklin et al., 2008), understanding the role of ovarian hormones and specifically, estrogens in maintaining nicotine use are essential.

Recently, the influence of ovarian hormones on drug-related behaviors has been of great interest (for review, see Knouse and Briand, 2021; Nicolas et al., 2022; Peart et al., 2022). To our knowledge, only two preclinical studies have explored the role of ovarian hormones in nicotine self-administration, only one of which was conducted outside our laboratory. One study found that 4 d of a high dose of 17β-estradiol (E2) administration following ovariectomy (OVX) increased nicotine intake in female rats (Flores et al., 2016). Our results showed that a lower dose E2 supplementation before nicotine self-administration acquisition partially restores OVX-induced suppression of nicotine consumption (Maher et al., 2021). Importantly, nicotine consumption levels only began to dissociate from OVX-vehicle levels after 14 d of daily supplementation with a physiologically relevant, tonically administered dose of E2. Thus, it remains unclear why E2 alone cannot completely restore nicotine consumption to ovary-intact levels when given at different doses.

Along with E2, estrone (E1) and estriol (E3) are the three primary circulating estrogens, with the latter most prevalent during pregnancy (Veler et al., 1930; Thayer et al., 1931; Huffman et al., 1940; Fuentes and Silveyra, 2019). In women, E2 and E1 are synthesized and released primarily from the ovary. All three estrogens bind to estrogen receptors (ERs), with E2 having the most affinity of the three (Kuhl, 1990), followed by E1 and, finally, E3. There are two ER isoforms, ERα (*Esr1* gene) and ERβ (*Esr2* gene), that are derived from different genes on separate chromosomes in humans, chromosome 6 and chromosome 14, respectively. While their genes are distantly located, their functions are similar as they are ligand-induced transcriptional factors and signaling molecules (Fuentes and Silveyra, 2019). Estrogens bind to the ERs, and then signal to DNA via binding to promoters of genes (Fuentes and Silveyra, 2019). Some reports have suggested inverse functions for ERα and ERβ, which might involve DNA sequences that they target, such as the UGT glucuronosyltransferases that metabolize hormones (Sundararaghavan et al., 2017). ERs also participate in protein-protein interactions that are involved in signaling cascades that regulate cells such as GABAergic medium spiny neurons (MSNs) within the nucleus accumbens core (NAcore). Interestingly, nicotine reduces circulating estrogens (Ruan and Mueck, 2015), and this may have a critical consequence on chronic nicotine users, which could be specific to a woman’s reproductive health. Combined, interactions between nicotine and estrogens and involvement of estrogens in the reward pathway supports a significant role of estrogens in nicotine use.

Nicotine signals via the nicotinic acetylcholine receptors (nAChRs) and through activating β2-containing nAChRs, can increase dopamine release from the ventral tegmental area (VTA) into the NAcore (Pontieri et al., 1996; Mansvelder and McGehee, 2002; Mansvelder et al., 2002; Grieder et al., 2019). Interestingly, positive allosteric modulators of nAChRs reverse desensitization caused by nicotine (Pauley, 2008; Jin and Steinbach, 2011), and E2 acts as an allosteric modulator of nAChRs (Jin and Steinbach, 2011). It has also been shown that OVX can reduce the density of nAChRs containing the α7 subunit in various brain regions, and estrogen replacement increases the binding of α-bungarotoxin (nAChR antagonist) at these receptors to control levels (Miller et al., 1982, 1984; Morley et al., 1983; Arimatsu et al., 1985; Koylu et al., 1997; Centeno et al., 2006), suggesting a restoration of these receptors. This is important because the α7 nAChRs impact glutamate transmission (Cheng and Yakel, 2015). However, in the context of nicotine self-administration, it is not currently known how estrogens impact nAChRs in the reward pathway in females.

Nicotine dysregulates glutamate homeostasis within the corticostriatal circuitry (Gipson et al., 2013b; Powell et al., 2019), altering GABAergic MSN functional and structural plasticity. In the absence of nicotine, E2 alters NAcore dendritic spine morphology, decreasing spine density and creating immature spine phenotypes (Staffend et al., 2011; Peterson et al., 2015), changes that are remarkably similar to those induced by repeated psychostimulant exposure (Dumitriu et al., 2012). E2 exposure exacerbates glutamate-induced neuronal damage in organotypic hippocampal culture (Sato et al., 2002), and locally synthesized E2 from aromatase modulates synaptic transmission and plasticity within the auditory cortex (Soutar et al., 2022), showing complex E2-glutamate interactions. Additionally, ERs are located on MSNs within the NAcore (Tonn Eisinger et al., 2018).

Taken together, it is biologically feasible that E2 impacts glutamatergic plasticity in MSNs within the reward pathway. For these reasons, here we examined the role of precipitous ovarian hormone loss via OVX and estrogen supplementation in mediating nicotine self-administration, mRNA expression profile of nAChRs and ERs in the NAcore and VTA, and NAcore MSN physiology. We found that ovarian hormones, explicitly estrogen, regulates glutamatergic synaptic plasticity in the NAcore in a nicotine-specific fashion.

## Materials and Methods

### Animals

**Figure 1.**
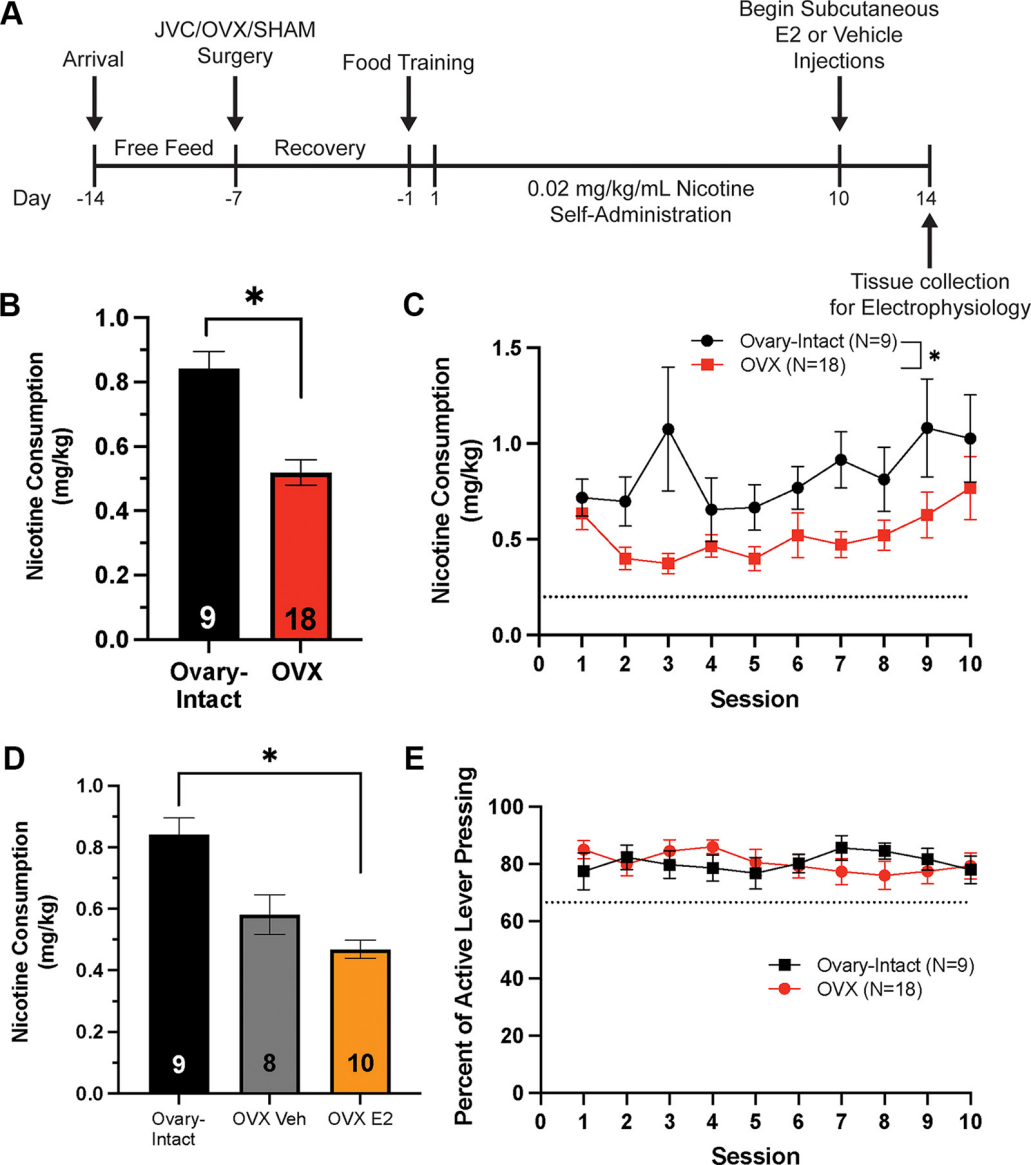
Ovarian hormones regulate nicotine consumption during acquisition of a low dose of nicotine. ***A***, A timeline of experimental procedures, including surgical procedures, self-administration, subcutaneous hormone/vehicle injections, and tissue collection for electrophysiology. ***B***, A main effect of group was found on nicotine consumption. Ovary-intact freely cycling females have more nicotine consumption than ovariectomized (OVX) females across sessions 1–10 (*p <* *0.05; mean ± SEM). Data presented as average consumption over all days. ***C***, Nicotine consumption across sessions 1–10. The dotted line in ***C*** represents the minimum consumption criterion of 0.2 mg/kg. ***D***, A main effect of group was found for nicotine consumption whereas OVX females selected to receive estrogen (E2) consumed significantly less nicotine than ovary-intact females across the first 10 sessions (*p < 0.05; mean ± SEM). Data presented as average consumption over all days. ***E***, The percentage of active lever presses (active divided by active+inactive × 100) across sessions is shown for OVX and ovary-intact groups. No significant main effect of group was found to effect lever discrimination (*p* > 0.05; mean ± SEM). The dotted line in ***E*** represents the 2:1 active:inactive lever press criterion (at 66.67%).*Figure Contributions:* Jonna M. Leyrer-Jackson performed the experiments. Joshua S. Beckmann analyzed the data.

**Figure 2.**
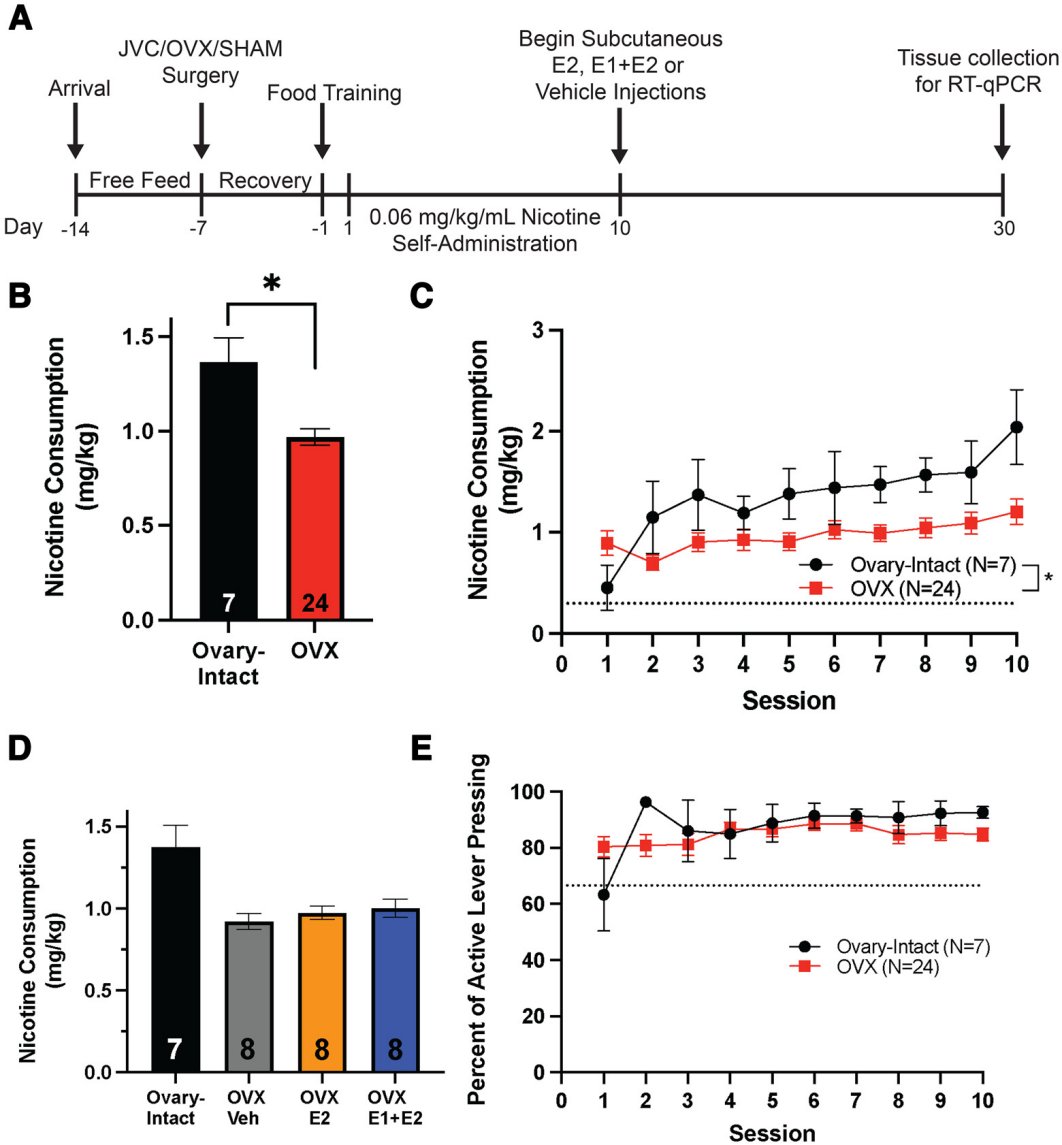
Ovarian hormones regulate nicotine consumption during acquisition at a high dose of nicotine. ***A***, A timeline of experimental procedures, including surgical procedures, self-administration, subcutaneous hormone/vehicle injections, and tissue collection for RT-qPCR. ***B***, A main effect of group was found on nicotine consumption and ovary-intact freely cycling females consumed more nicotine than OVX females across sessions 1–10 (*p <* *0.05; mean ± SEM). Data presented as average consumption over all days. ***C***, Nicotine consumption in ovariectomized (OVX) and ovary-intact females across sessions 1–10 (*p < 0.05; mean ± SEM). The dotted line in ***C*** represents the minimum consumption criterion of 0.3 mg/kg. ***D***, No main effect of subgroup was found on nicotine across the first 10 sessions (*p* > 0.05; mean ± SEM). Data presented as average consumption over all days. ***E***, There was no main effect of group on lever pressing discrimination (active divided by active+inactive × 100) across sessions is shown for OVX and ovary-intact groups (mean ± SEM). The dotted line in ***E*** represents the 2:1 active:inactive lever press criterion.*Figure Contributions:* Erin E. Maher, Shailesh Khatri, and Emma Bondy performed the experiments. Erin E. Maher analyzed the data.

Two- to three-month-old female Sprague Dawley rats (*n* = 115; 225–300 g; Charles River) were singly housed on a 12-h reverse light cycle and had access to food and water *ad libitum* before experimental procedures. All animals were kept in a humidity-controlled and tempera ture-controlled animal facility and were handled daily. All practices and procedures were approved by the Arizona State University and University of Kentucky Institutional Animal Care and Use Committee (IACUC). A timeline of the experimental procedures discussed below is outlined in Figures 1*A* and 2*A*. Twenty-four total rats were omitted from the study because of loss of catheter patency. Data from these animals was not included in any analysis. Some of the control groups received sham surgery to compare ovary-intact to OVX females in the context of nicotine self-administration. For electrophysiological endpoints (experiment 1), nicotine-naive females were not given sham surgery, but were left ovary-intact. For RT-qPCR endpoints (experiment 2), all rats underwent OVX with or without nicotine self-administration.

### Surgical procedures

#### Jugular vein catheter

All rats used for self-administration were anesthetized with ketamine HCl (80–100 mg/kg, i.m.) and xylazine (8 mg/kg, i.m.) and underwent surgical implantation of intravenous jugular catheters (*n* = 109) as described in our previous publications (Gipson et al., 2013a; Maher et al., 2021). Directly following jugular vein catheterization, a portion of animals underwent either OVX (*n* = 78) or sham surgery (*n* = 31). Rats were given cefazolin (100 mg/kg, i.v.), heparin (10 usp, i.v.), and meloxicam (1 mg/kg, i.v.) on the day of surgery and once daily for 3 d during the postsurgical recovery period. Heparin was administered throughout the postsurgical recovery period and throughout nicotine, self-administration to maintain catheter patency. Catheter patency was checked throughout self-administration experiments using Brevital (3 mg/kg, i.v.), which induces and maintains anesthesia for 2–5 min. Females received sham surgery to determine whether the process surgery itself altered nicotine self-administration. Additionally, a small portion of animals (*n* = 34) underwent OVX without jugular vein catheterization. These animals served as a nicotine naive group to explore potential nicotine-specific effects on intrinsic electrophysiological characteristics of NAcore MSNs as well as mRNA translation of ERs and nAChRs.

#### OVX

All rats that underwent OVX or sham surgery were anesthetized with ketamine HCl (80–100 mg/kg, i.m.) and xylazine (8 mg/kg, i.m.). A dorsolateral incision through the skin and peritoneum was made directly above both ovaries. A ligature was then applied to the tip of each uterine horn, and each ovary was removed. For sham surgeries, the incision was made through the skin and peritoneum; however, the ovaries were left intact. The abdominal wall muscle was sutured using coated VICRYL suture (Ethicon), and staples were used to adhere the skin incision above the muscle suturing. Rats were given cefazolin (100 mg/kg, s.c.) and meloxicam (1 mg/kg, s.c.) on the day of surgery and once daily for 3 d during the postsurgical recovery period.

### Operant conditioning chambers

All food training and nicotine self-administration procedures were conducted in 28 modular test chambers (13 ENV-008, 15 ENV-007; Med Associates). All operant chambers used have been previously described (Overby et al., 2018). Each chamber contained two levers, separated by a flanked pellet receptacle, which was used for food training. Two stimulus lights, one above each lever, were used as “cue” lights and paired with active lever pressing during nicotine self-administration sessions. The right lever was designated as the “active” lever for both food training and nicotine self-administration, which yielded either one food pellet or one intravenous nicotine infusion, respectively.

### Food training procedures

After 6 d postsurgery, rats were food restricted to 15–20 g of chow per day. Food restriction occurred a minimum of 2 h before the food training session was initiated and was maintained throughout the remainder of the experiment, including throughout self-administration. Rats underwent a 15 h food training session, where one active lever press [fixed ratio-1 (FR-1) schedule of reinforcement] resulted in the delivery of one food pellet (45 mg/pellet; Bio Serv). Food pellet delivery was not paired with any discrete cues. Rats were required to obtain an active to inactive lever press ratio of 2:1 throughout food training and were required to accumulate a minimum of 200 active lever presses throughout the session. Animals underwent additional food training sessions if these criteria were not met. Additional food training was required for 10 out of 60 animals (10%).

### Nicotine self-administration

Rats began nicotine self-administration sessions 24 h following their final food training session, between 8 and 10 d following OVX. Rats were trained to self-administer either a low (0.02 mg/kg/infusion; experiment 1) or high (0.06 mg/kg/infusion; experiment 2) dose of intravenous nicotine on an FR-1 schedule of reinforcement which was the same schedule used in food training. Two doses of nicotine were tested to determine whether the effects of E2 on nicotine consumption were dose-dependent, as we have previously shown that estrogens can impact consumption outcomes when the dose is manipulated (Maher et al., 2021). Nicotine was delivered over a 5.9-s period following an active lever press. Each active lever press was paired with a concurrent nicotine infusion, cue lights, as well as a high-frequency tone (2900 Hz), followed by a 20-s timeout period, during which additional active lever presses were recorded but yielded no additional infusions, cues, or programmed consequences. The inactive lever was presented at all times, and pressing was recorded but did not produce programmed consequences. All sessions were 2 h in duration. All animals were required to complete 10 sessions of nicotine SA before receiving subcutaneous hormone injections. For animals receiving a low dose of nicotine, subcutaneous injections of E2 (3 μg/0.1 ml; made in sesame oil) or vehicle (0.1 ml sesame oil) were given 2 h before self-administration sessions for four consecutive days. This injection schedule was chosen to mimic similar studies (Flores et al., 2016, 2020) and to avoid potential changes in the cholinergic system known to occur with chronic supplementation (Lapchak et al., 1990). This dosage of E2 was chosen based on previous work demonstrating that it produces E2 levels similar to ovary-intact females (Long et al., 2019; Kirshner et al., 2020; Koebele et al., 2020). Vaginal smears were performed after the final 5 d of nicotine self-administration to confirm successful OVX and hormone treatment in E2-treated and E1+E2-treated rats. Of the animals that began injections, 12/31 OVX and 11/20 ovary-intact animals did not complete the four continuous injections because of loss of catheter patency. For animals receiving a high dose of nicotine, subcutaneous injections of E2 (3 μg/0.1 ml; made in sesame oil), E1+E2 (10 μg E1 + 3 μg E2/0.1 ml; made in sesame oil), or vehicle (0.1 ml sesame oil) were given 2 h before self-administration sessions for 20 consecutive days. This schedule was chosen to determine the effect of chronic hormone replacement on nicotine intake, and the dose of E1 was chosen based on previous studies that administered subcutaneous E1 in rats and studied its effects in the brain (Knoll et al., 2000). Immediately following the last self-administration session for the low dose group, animals were anesthetized and killed for whole-cell electrophysiology, and for the high-dose group, animals were killed for RT-qPCR.

### Electrophysiological recordings

In experiment 1, animals were killed following nicotine self-administration and 4 d of hormone treatments. Rats were anesthetized with CO_2_ and rapidly decapitated, similar to other studies using whole-cell electrophysiology (Dubé et al., 2005; Spindle and Thomas, 2014; Leyrer-Jackson et al., 2020). Brains were removed and placed into ice-cold cutting solution (cutting aCSF) containing (in mmol/l): NaCl, 120; NaHCO_3,_ 25; dextrose, 10; KCl, 3.3; NaH_2_PO_4_, 1.23; CaCl_2_, 1.8; MgCl_2_, 2.4 and saturated with carbogen (95% O_2_/5% CO_2_). Solution pH was adjusted to 7.40 ± 0.03, and osmolarity was adjusted to 295 ± 5 mOsm. Coronal slices containing the NAcore were made on a vibrating tissue slicer (Leica, VT1000S) at 300 μm thick. Slices were cut in cutting aCSF and immediately transferred to a recording chamber filled with recording aCSF solution containing (in mmol/l): NaCl, 120; NaHCO_3_, 25; KCl, 3.3; NaH_2_PO_4_, 1.23; CaCl_2_, 0.9; MgCl_2_, 2.0; dextrose, 10, osmolarity adjusted to 295 ± 5 mOsm and pH adjusted to 7.40 ± 0.03. The holding chamber was continuously oxygenated with a carbogen and incubated at 34°C for 45 min before being allowed to cool to room temperature before recordings. Tissue slices were then transferred to a recording chamber and continuously perfused at a flow rate of 1–2 ml/min with recording aCSF. MSNs were visually identified on an Olympus BX51WI microscope using infrared DIC microscopy. Whole-cell recordings were made from the soma of MSNs using recording pipettes (7–15 mΩ) after establishing a giga-ohm seal (resistance range: 1–10 GΩ). Recording pipettes were filled with an intracellular solution containing (in mmol/l): K-gluconate, 135; NaCl, 12; K-EGTA, 1; HEPES, 10; Mg-ATP, 2 and tris-GTP, 0.38. Osmolarity was adjusted to 285 ± 5 mOsm, and pH was adjusted to 7.30 ± 0.01. Upon membrane rupture, cell membrane potential was held at –80 mV. Only cells that exhibited overshooting, thin action potentials, normal resting membrane potentials, and changes in uncompensated access resistance less than 20 mΩ were included in analyses. Access resistance, membrane resistance, and resting membrane potential (monitored in zero current mode) were continuously monitored throughout recordings to ensure cell viability. In addition to these measures, input-output curves were generated using a 15-step voltage injection protocol (administered by the recording pipet) ranging from –35 to 105 mV to measure cellular output. Given that prelimbic fibers densely target the NAcore within the dorsal region (Gipson et al., 2013a,b; Stefanik et al., 2016; Neuhofer and Kalivas, 2018), a stimulating electrode was placed in the dorsal region of the NAcore to activate primarily prelimbic glutamatergic fibers. However, it cannot be completely ruled out that other glutamatergic fibers were activated because of the nonspecificity of electrical stimulation. AMPA currents, evoked by electrical stimulation, were first evoked at –80 mV. The membrane potential was gradually increased to +40 mV, where EPSCs, composed of both AMPA and NMDA receptor-mediated currents, were obtained. DNQX (20 μm) was then bath applied for 5 min, and NMDA receptor-mediated currents were then elicited at +40 mV. AMPA currents were then obtained by subtracting the NMDA receptor-mediated current, taken at +40 mV, from the whole EPSC, measured at +40 mV. AMPA/NMDA ratios were calculated by measuring the peak amplitude of each current measured at +40 mV and taking the ratio. All recordings were conducted in an intact network, where GABA receptor antagonists were not contained within the recording solution. Thus, intact inhibitory currents could alter the results presented in the current manuscript. The recording software Axograph was used for conducting recordings. All recordings were digitized at 10 kHz and saved using the digidata interface (Molecular Devices), and analyzed offline using Axograph.

### RT-qPCR

#### Tissue collection

In experiment 2, animals were rapidly decapitated immediately following the conclusion of their last day of nicotine self-administration. Brains were quickly dissected, and coronal sections (2 mm thick) were cut from the native brain tissue using a rat brain matrix (Harvard Apparatus). Sections containing the NAcore were collected starting 7 mm caudal to the frontal pole and were collected with a 2-mm diameter tissue puncher (Miltex). Sections containing the VTA were collected starting 13 mm caudal to the frontal pole, and the VTA was collected from both hemispheres using a 1.5-mm diameter tissue puncher (Miltex). On the first coronal sections, TTC staining was performed, and brain tissue from the border of the lesion was dissected using a punch biopsy needle. Tissue samples were stored at −80°C until homogenization.

#### RNA isolation and cDNA synthesis

Measurement of gene expression via RT-qPCR was performed using our previously described methods (Creeden et al., 2022). RNA was isolated using a QIAzol lysis reagent (QIAGEN or Invitrogen) using a QIAGEN TissueLyser LT (QIAGEN) with a setting of 50 oscillations per second for 6 min. RNA was then extracted from samples using the miRNeasy Mini kit (QIAGEN). RNA concentration and purity were assessed using a NanoDrop One^C^ spectrophotometer (Thermo Fisher Scientific), and cDNA was synthesized using High Capacity cDNA Reverse Transcription kit (Applied Biosystems) and 1 μg of RNA. PCR amplification of genomic targets was performed by qualitative RT-qPCR (qt-RT-PCR) using TrueAmp SYBR Green qPCR SuperMix. Esr1, F, CACACACGCTCTGCCTTGAT and R, CTGCTGGTTCAAAAGCGTCT; Esr2, F, GGAGGTGCTAATGGTGGGAC and R, CCCTCATCCCTGTCCAGAAC; Chrnb2, F, CCAACTCAATGGCGCTGTTC and R, GCTCCTCTGTGTCAGTTCCC, Chrna7, F, GTCCTGGTCCTATGGAGGGT and R, GATCCCATTCTCCGTTGGGG. Normalization was performed in separate reactions with primers to 36B4 (F, GAACATCTCCCCCTTCTCCTTC and R, ACCTCTGGGCTGTAGATGCT), an endogenous housekeeping gene.

### Immunohistochemistry

For confocal microscopy immunostaining, one female rat was deeply anesthetized with an overdose of euthasol (excess of 0.25 ml/kg i.p.) and transcardially perfused with 0.01 m PBS (2–3 min) followed by 300 ml of a fixative solution containing 4% paraformaldehyde. Subsequently, the brain was sectioned at 60 μm using a vibratome. Free-floating sections containing the VTA and NAcore were selected and were incubated in a blocking solution of 1%BSA-PBS for 30 min. To determine whether ERs are present on dopaminergic neurons in the VTA, VTA sections were incubated in three primary antibodies (anti-ERα, anti-ERβ, and anti-TH) that were diluted in 0.01 m PBS containing 1% BSA, 0.3% triton and 0.05% sodium azide for 24 h at room temperature on a shaker. Sections were then rinsed and incubated in the secondary antibodies conjugated to Alexa Fluor 488, Alexa Fluor 594, and Alexa Fluor 647 for 2 h. Sections were mounted onto glass slides and coverslipped using an anti-fade mounting medium. To determine whether ERs are located on neurons in the NAcore, NAcore sections were incubated in two primary antibodies (anti-ERα, anti-ERβ) that were diluted in 0.01 m PBS containing 1% BSA, 0.3% triton, and 0.05% sodium azide for 24 h at room temperature on a shaker. Sections were then rinsed and incubated in the secondary antibodies conjugated to Alexa Fluor 594 and Alexa Flour 647 for 2 h. A full description of antibodies used in this study can be found in Table 1. In order to identify cell bodies in the NAcore, sections were mounted onto glass slides and coverslipped using an anti-fade mounting medium that contained DAPI. All confocal micrographs were captured using a Nikon A1R Inverted Confocal Microscope. Please see Table 1 for information regarding antibodies and dilutions.

**Table 1:** Antibodies and dilutions for immunohistochemistry

Antibody name	Immunogen	Antibody info	Dilution
Anti-tyrosine hydroxylase (TH)	Two synthetic peptide/keyhole limpet hemocyanin (KLH) conjugates	Abcam; catalog #AB76442; RRID:AB_1524535; chicken (polyclonal)	1:2000
Anti-estrogen receptor α (ERα)	Synthetic peptide; this information is proprietary to Abcam and/or its suppliers	Abcam; catalog #AB209288; RRID:AB_2757814; rabbit (monoclonal)	1:1500
Anti-estrogen receptor β1 (ERβ)	Recombinant fragment corresponding to human estrogen receptor β1 (C terminal)	Abcam; catalog #AB187291; RRID: AB_2757815; mouse (monoclonal)	1:1500
Secondary antibodies			
Alexa Flour 488 anti-chicken IgG	n/a	ThermoFisher Scientific; catalog #A-11039	1:500
Alexa Flour 594 anti-rabbit IgG	n/a	ThermoFisher Scientific; catalog #A-11012	1:500
Alexa Flour 647 anti-mouse IgG	n/a	ThermoFisher Scientific; catalog #A-21235	1:500

### Drugs

(-)Nicotine tartrate (MP Biomedicals) was dissolved in sterile 0.9% saline, and pH was adjusted to 7.2–7.4 using 1 m NaOH. E2 was purchased from Sigma-Aldrich and diluted to 3 μg/0.1 ml in sesame oil (Sigma-Aldrich). E1 was purchased from Sigma and was diluted to 10 μg/0.1 ml in 3 μg/0.1 ml E2 in sesame oil (Sigma-Aldrich). 6,7-dinitroquinoxaline-2,3(1H,4H)-dione (DNQX) was purchased from Tocris Biotechne and diluted to a stock concentration of 20 mm in dimethyl sulfoxide (DMSO). Before recordings, DNQX was diluted to a working concentration of 20 μm in recording aCSF.

### Data analysis

Animals that lost catheter patency were omitted from data analysis. The JMP software from SAS was used to conduct linear mixed-effects (LME) modeling on self-administration data, including nicotine consumption and the percent of active lever pressing. Session was treated as a continuous factor, group (OVX vs sham) or subgroup (vehicle, E2, or E1+E2) were treated as nominal factor, and subject was treated as a random factor. For electrophysiological data, LME modeling was conducted separately for each measurement (cellular capacitance, input/output curves, resting membrane potential, and AMPA/NMDA ratio). For RT-qPCR data, LME modeling was conducted separately for each gene. Tukey’s *post hoc* tests were conducted where appropriate using an α set to 0.05. All values are presented as mean ± SEM. Specific analyses used are presented within the results and figure legends where appropriate.

## Results

### Nicotine self-administration

#### Nicotine acquisition phase (sessions 1–10)

Before nicotine acquisition, all animals passed the criteria for food training procedures, and LME analysis revealed no difference in the percent of active lever presses between ovary-intact sham rats and OVX rats (*F*_(1,28)_ = 0.97, *p *>* *0.05). Nicotine self-administration experiments were completed for both low (0.02 mg/kg/infusion; experiment 1) and high (0.06 mg/kg/infusion; experiment 2) doses of nicotine (see Figs. 1*A*, 2*A* for experimental timelines for the two experiments). For the first 10 sessions of self-administration for both low and high doses, nicotine consumption and the percent of active lever presses were compared across all groups (either ovary-intact-vehicle, OVX-vehicle, OVX-E2, and OVX-E1+E2 for high nicotine dose, or ovary-intact, OVX-vehicle, or OVX-E2 for low nicotine dose). LME analysis in the low nicotine dose experiment revealed a significant main effect of session (*F*_(1,24)_ = 8.75, *p *<* *0.05; Fig. 1*C*), indicating that nicotine consumption increased across sessions for all groups. Additionally, LME analysis revealed a significant main effect of group (*F*_(2,24)_ = 7.38, *p *<* *0.05; Fig. 1*B*), indicating that overall lever pressing differed across groups. A follow-up Tukey’s HSD *post hoc* test on the main effect of group indicated that OVX females subsequently assigned to the E2 group (for sessions 11–14) self-administered significantly less nicotine than ovary-intact females during sessions 1–10 (*F*_(2,24)_ = 3.49, *p *<* *0.05; Fig. 1*D*). In the high nicotine dose experiment, LME modeling also revealed a significant main effect of session (*F*_(1,29)_ = 24.27, *p* < 0.05; Fig. 2*C*) as well as a main effect of group (*F*_(1,29)_ = 4.37, *p *<* *0.05; Fig. 2*B*). Tukey’s HSD *post hoc* test on the main effect of group indicated no significant main effect of eventual treatment groups of OVX animals on nicotine consumption (*F*_(3,27)_ = 1.36, *p *>* *0.05; Fig. 2*D*). Thus, because of the lack of baseline differences, all OVX groups were combined for analysis of sessions 1–10 and are referred to as the OVX group for acquisition sessions. Next, the percent of active lever presses [active/(active+inactive) × 100] between ovary-intact and OVX groups were evaluated for both low and high doses of nicotine. LME analysis revealed no main effect of group or session on lever discrimination for both the low (*p *>* *0.05; Fig. 1*E*) and high (*p *>* *0.05; Fig. 2*E*) nicotine dose experiments. These results indicate that both ovary-intact and OVX female rats readily discriminated between the active and inactive levers during initial nicotine self-administration sessions at both the 0.02 and 0.06 mg/kg/infusion nicotine doses Together, these results indicate that OVX females acquired nicotine self-administration but consumed significantly less nicotine than ovary-intact, freely cycling females for both low and high doses of nicotine during acquisition.

#### Nicotine maintenance and hormone treatment phase [sessions 11–14 (experiment 1) or 11–30 (experiment 2)]

**Figure 3.**
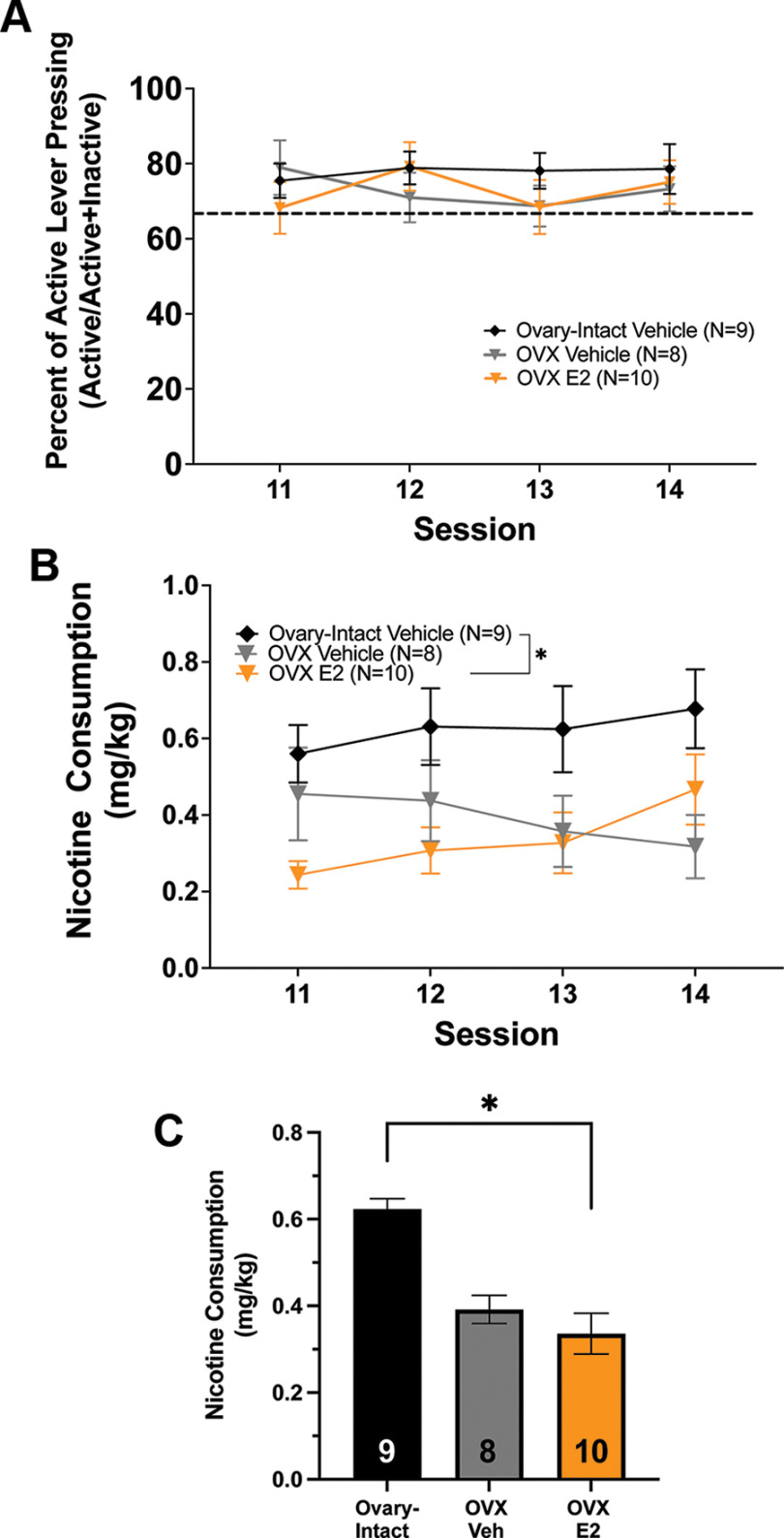
Subchronic estrogen (E2) treatment does not restore ovariectomy (OVX)-induced suppression of low-dose nicotine consumption. ***A***, No main effect of group on percentage of active lever presses (active divided by active+inactive × 100) across sessions was found for sessions 11–14 (*p* > 0.05; mean ± SEM). The dotted line represents the 2:1 active:inactive lever press criterion. ***B***, Nicotine consumption across sessions 11–14 (mean ± SEM). No main effect of session was found on nicotine consumption (*p* > 0.05; mean ± SEM). ***C***, A main effect of subgroup on nicotine consumption was found in which ovary-intact vehicle-treated females consumed significantly more nicotine than OVX-E2 but not OVX-vehicle (Veh) females during sessions 11–14 (*p <* *0.05; mean ± SEM). Data presented as average consumption over all days.*Figure Contributions:* Jonna M. Leyrer-Jackson performed the experiments. Joshua S. Beckmann analyzed the data.

In experiment 1, OVX females were given four sessions of either vehicle or E2 treatment following acquisition, during sessions 11–14. For sessions 11–14, LME analysis revealed no significant main effect of group or session on percent of active lever presses ([active/(active+inactive) × 100; Fig. 3*A*, *p* > 0.05]. For rats receiving a low dose of nicotine, consumption across sessions 11–14 was compared between intact-vehicle, OVX-vehicle, and OVX-E2 female rats using LME modeling. LME analysis revealed no significant effect of session on nicotine consumption (*p* > 0.05; Fig. 3*B*). LME analysis also revealed a significant main effect of group on treatment (*F*_(2,24)_ = 3.54, *p *<* *0.05; Fig. 3*C*). Tukey’s HSD *post hoc* test on this effect shows that ovary-intact females had a significantly higher number of infusions than OVX-E2 females but not OVX-vehicle females during sessions 11–14 (*F*_(24)_ = 2.5, *p *<* *0.05; Fig. 3*C*,*B*). Thus, 4 d of tonic E2 treatment did not elevate nicotine consumption back to ovary-intact levels at the lower nicotine dose.

**Figure 4.**
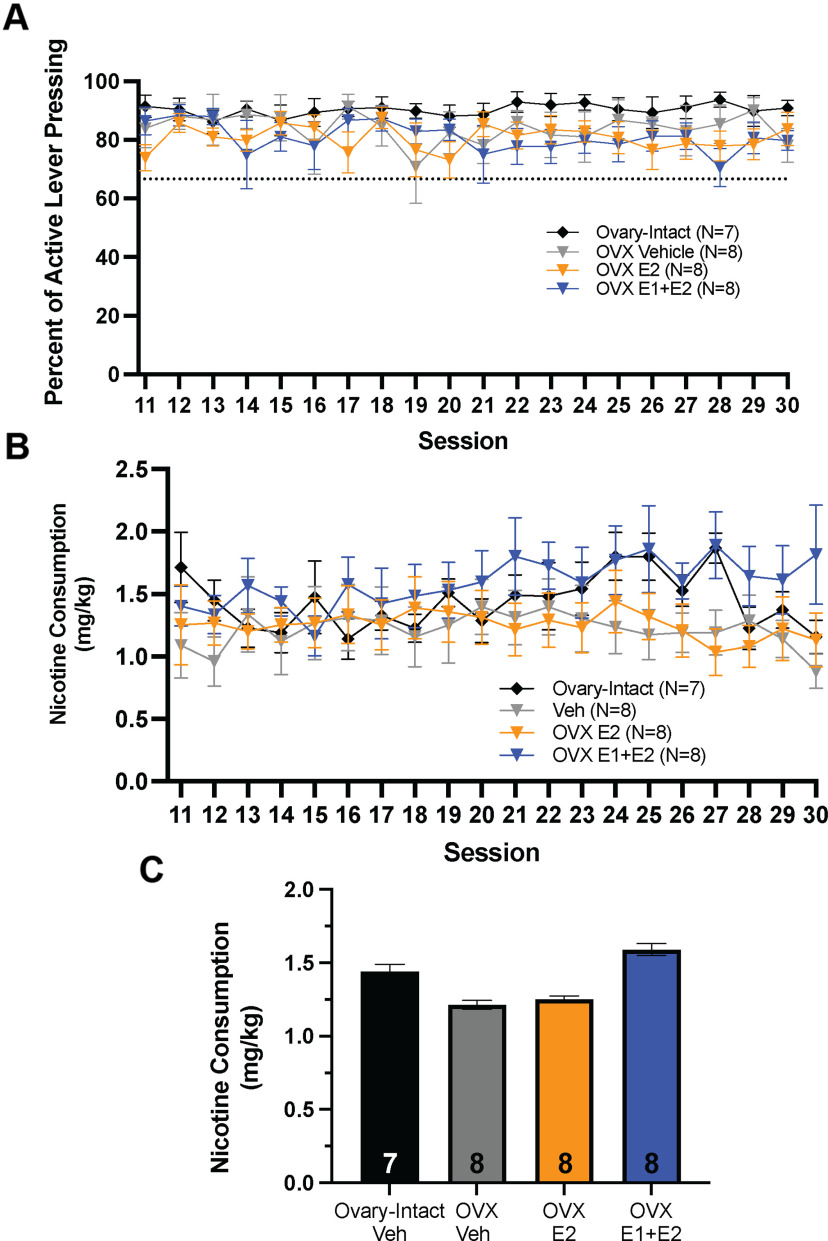
Chronic hormone treatment does not restore ovariectomy (OVX)-induced suppression of high-dose nicotine consumption. ***A***, There was no main effect of session on discrimination of active lever presses (active divided by active+inactive × 100) across sessions is shown for OVX-estrogen(E2), OVX-estrone (E1)+E2, OVX-vehicle (Veh), and ovary-intact-vehicle groups during sessions 11–30 (*p* > 0.05; mean ± SEM). The dotted line represents the 2:1 active:inactive lever press criterion (mean ± SEM). ***B***, There was no main effect of group on nicotine consumption across sessions 11–30 (*p* > 0.05; mean ± SEM). ***C***, There was no main effect of subgroup on nicotine consumption between ovary-intact-vehicle, OVX-Veh, OVX-E2, or OVX-E1+E2 females during sessions 11–30 (*p *>* *0.05; mean ± SEM). Data presented as average consumption over all days.*Figure Contributions:* Erin E. Maher, Shailesh Khatri, and Emma Bondy performed the experiments. Erin E. Maher analyzed the data.

In the second experiment, rats received longer hormone treatments and a higher dose of nicotine than in experiment 1, whereby all rats received 20 nicotine self-administration sessions with hormone or vehicle treatments following the acquisition phase. For sessions 11–30, LME analysis of the percent of active lever presses [active/(active+inactive) × 100] between ovary-intact-vehicle, OVX-vehicle, OVX-E2, and OVX-E1+E2 groups revealed no significant main effect of group (*p > *0.05; Fig. 4*A*). Next, consumption was compared between groups across sessions. LME analysis revealed no main effect of session (*F*_(1,26)_ = 0.81, *p > *0.05; Fig. 4*B*) or group (*F*_(1,26)_ = 1.15, *p*s > 0.05; Fig. 4*C*) on nicotine consumption across sessions 11–30.

### ER expression in the NAcore and VTA

**Figure 5.**
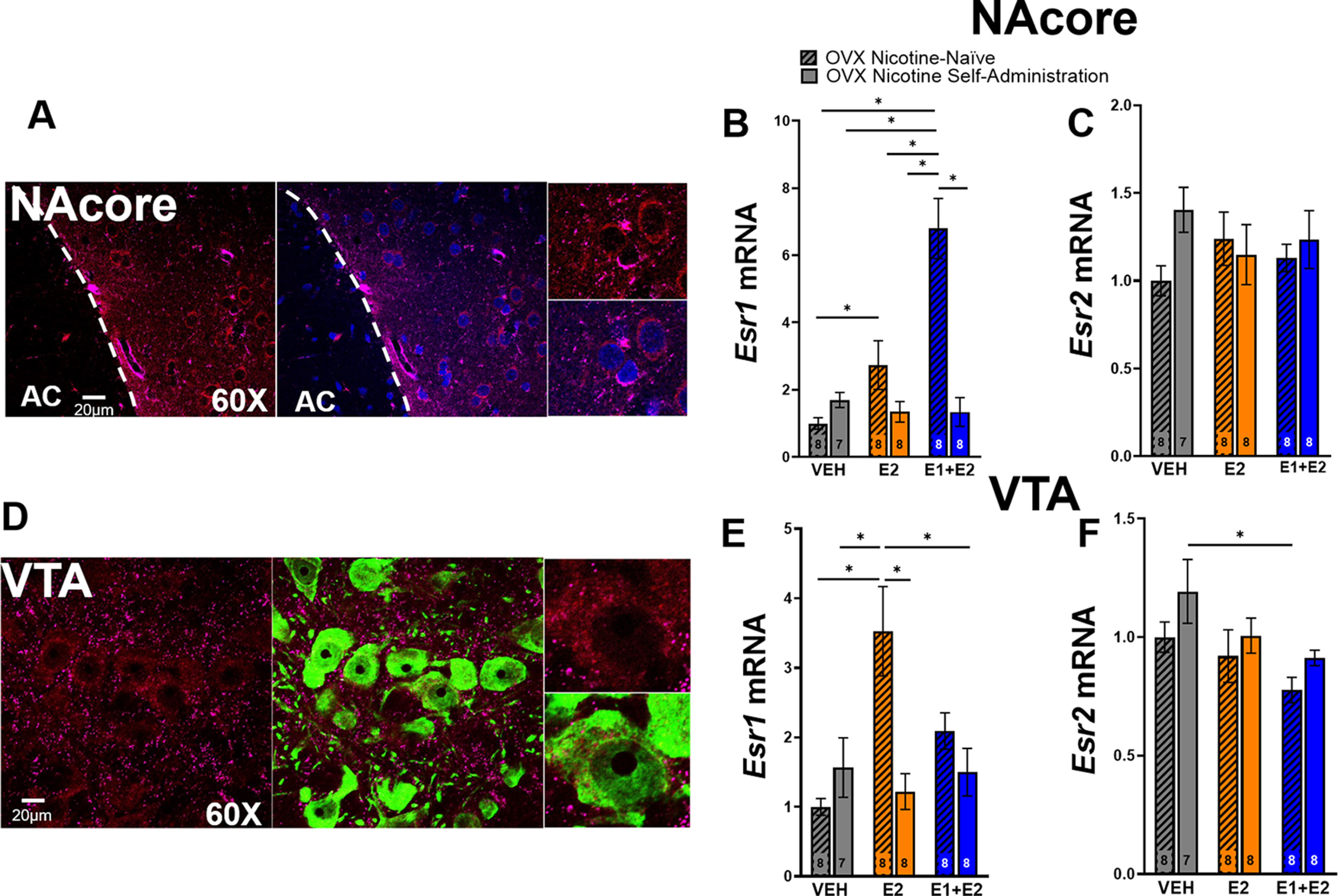
Nicotine consumption suppresses estrogen-induced increases in estrogen receptor alpha (ERα) mRNA in the nucleus accumbens core (NAcore) and ventral tegmental area (VTA) in ovariectomized (OVX) Females. ***A***, Immunocytochemistry reveals ERα (red) and estrogen receptor beta (ERβ) (purple) on somas (blue) in the NAcore (AC = anterior commissure, delineated by which dotted line). ***B***, RT-qPCR revealed a amin effect of group on *Esr1* (ERα) mRNA in the NAcore whereas nicotine naive estrone (E1)+estrogen (E2)-treated rats had higher levels compared with all other groups (*p < 0.05; mean ± SEM). ERα mRNA was also found to be higher in the nicotine naive E2-treated group compared with the nicotine naive vehicle (VEH) group (*p <* *0.05, mean ± SEM). ***C***, There was no main effect of group on *Esr2* (ERβ) in the NAcore between groups (LME; *p* > 0.05; mean ± SEM). ***D***, Immunocytochemistry reveals ERα (red) and ERβ (purple) on dopaminergic somas (green) in the VTA. ***E***, RT-qPCR revealed a main effect of group on ERα mRNA in the VTA. Nicotine naive females treated with E2 had higher mRNA compared with nicotine-naive vehicle, nicotine-experienced vehicle, nicotine-experienced E2, and nicotine-experienced E1+E2 (*p < 0.05; mean ± SEM). ***F***, RT-qPCR revealed a main effect of group on ERβ mRNA. Nicotine-experienced vehicle had more mRNA compared with nicotine naive E1+E2-treated animals (*p <* *0.05; mean ± SEM).*Figure Contributions:* Erin E. Maher collected the confocal micrographs. Erin E. Maher and Zachary A. Kipp performed RNA extraction and RT-qPCR experiments. Erin E. Maher analyzed the data.

Immunocytochemical labeling of the NAcore revealed both ERα and ERβ to be present on cell somas (Fig. 5*A*). Tissue from the NAcore and VTA was collected from OVX females in experiment 2 for RT-qPCR analysis. LME analysis was used to assess differences in ER isoform mRNA expression, which revealed a main effect of group on mRNA expression (*F*_(5,40)_ = 16.3, *p *<* *0.05; Fig. 5*B*). Tukey’s HSD *post hoc* test revealed that ERα mRNA was significantly increased in the NAcore of nicotine-naive animals treated with E1+E2 as compared with all other groups, and nicotine-naive E2-treated females had significantly higher ERα mRNA expression in the NAcore as compared with nicotine-naive vehicle-treated females. LME analysis showed no main effect of group on ERβ mRNA expression in the NAcore (*p *>* *0.05; Fig. 5*C*). Immunocytochemical labeling also revealed that ERα and ERβ are present on the somas of TH-positive neurons in the VTA, indicating that these receptors are localized on dopamine-producing neurons (Fig. 5*D*). In addition, LME analysis revealed a main effect of group on ERα mRNA in the VTA (*F*_(5,41)_ = 6, *p *<* *0.05; Fig. 5*E*). Tukey’s HSD *post hoc* test revealed nicotine-naive rats treated with E2 had significantly more ERα mRNA in the VTA than nicotine-naive and nicotine-experienced vehicle-treated groups, nicotine-experienced E2-treated animals, and nicotine-experienced E1+E2-treated rats. Finally, LME revealed a main effect of group on ERβ mRNA in the VTA (*F*_(5,41)_ = 2.63, *p *<* *0.05; Fig. 5*F*). Tukey’s HSD *post hoc* test showed that females that underwent nicotine self-administration with vehicle treatment had higher levels of ERβ mRNA as compared with nicotine-naive E1+E2-treated OVX females.

### nAChR expression in the NAcore and VTA

**Figure 6.**
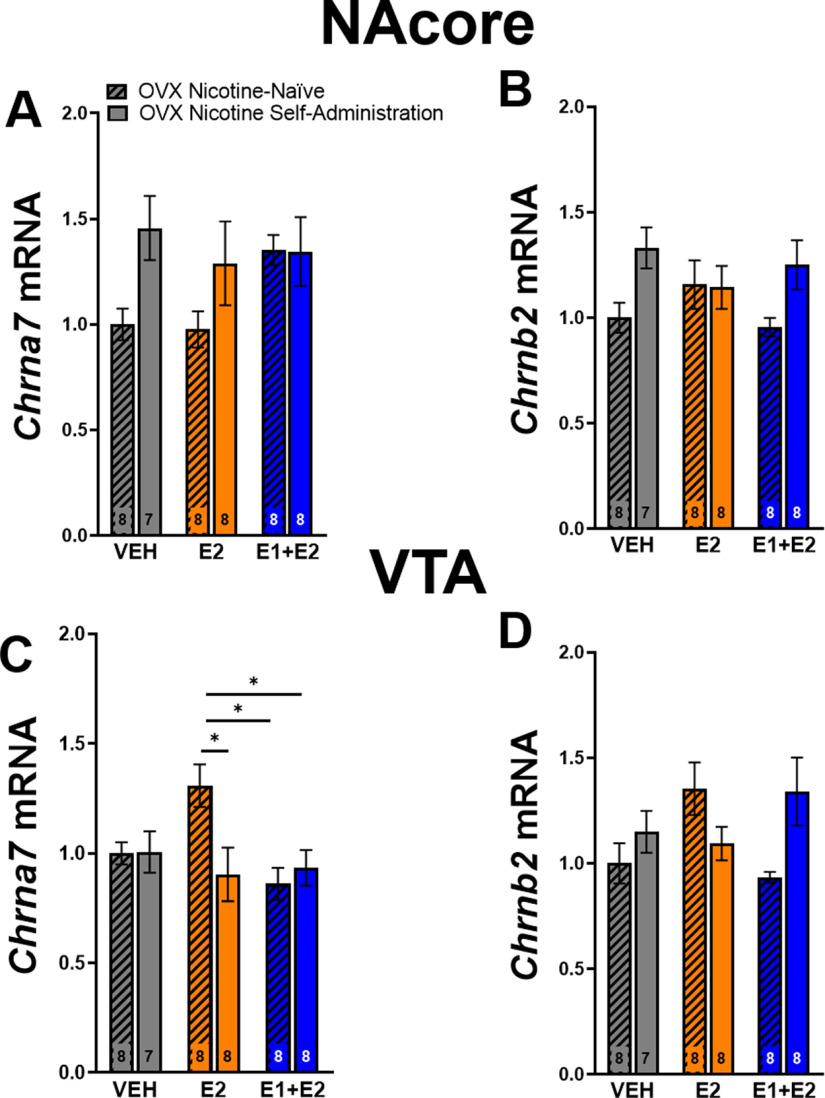
Nicotine consumption suppresses estrogen (E2)-driven nicotinic acetylcholine receptor (nAChR) α7 subunit mRNA synthesis in the ventral tegmental area (VTA) in ovariectomized (OVX) Females. ***A***, RT-qPCR revealed no main effect of group on α7 mRNA in the nucleus accumbens core (NAcore) (*p* > 0.05; mean ± SEM). ***B***, No main effect of group was found on nAChR β2 subunit mRNA between groups in the NAcore (*p* > 0.05; mean ± SEM). ***C***, A main effect of group was found on nAChR α7 subunit mRNA in the VTA whereas nicotine-naive rats treated with E2 had increased nAChR α7 subunit mRNA compared with nicotine-experienced E2-treated animals, nicotine-experienced estrone (E1)+E2-treated animals, and nicotine-naive E1+E2-treated animals (*p < 0.05; mean ± SEM). ***D***, No main effect of group was found in nAChR β2 subunit mRNA in the VTA were found between groups (LME; *p* > 0.05; mean ± SEM).*Figure Contributions:* Zachary A. Kipp and Genesee J. Martinez performed the RT-qPCR experiments. Erin E. Maher analyzed the data.

Tissue from the NAcore and VTA was collected from OVX females in experiment 2 for measurement of α7 and β2 nAChR mRNA via RT-qPCR. LME analysis revealed no main effect of group on α7 or β2 nAChR subunit mRNA in the NAcore (*p *>* *0.05; Fig. 6*A*,*B*). However, LME revealed a main effect of group on α7 nAChR mRNA in the VTA (*F*_(5,41)_ = 3.3, *p *<* *0.05; Fig. 6*C*). Tukey’s HSD *post hoc* test showed that nicotine-naive rats treated with E2 had significantly more VTA α7 nAChR subunit mRNA than nicotine-experienced E2-treated rats, nicotine-naive E1+E2-treated rats, and nicotine-experienced E1+E2-treated rats. Finally, LME analysis revealed no main effect of group on β2 nAChR subunit mRNA in the VTA between groups (*p *>* *0.05; Fig. 6*D*).

### Cessation of ovarian hormones potentiates NAcore MSNs after nicotine self-administration

**Figure 7.**
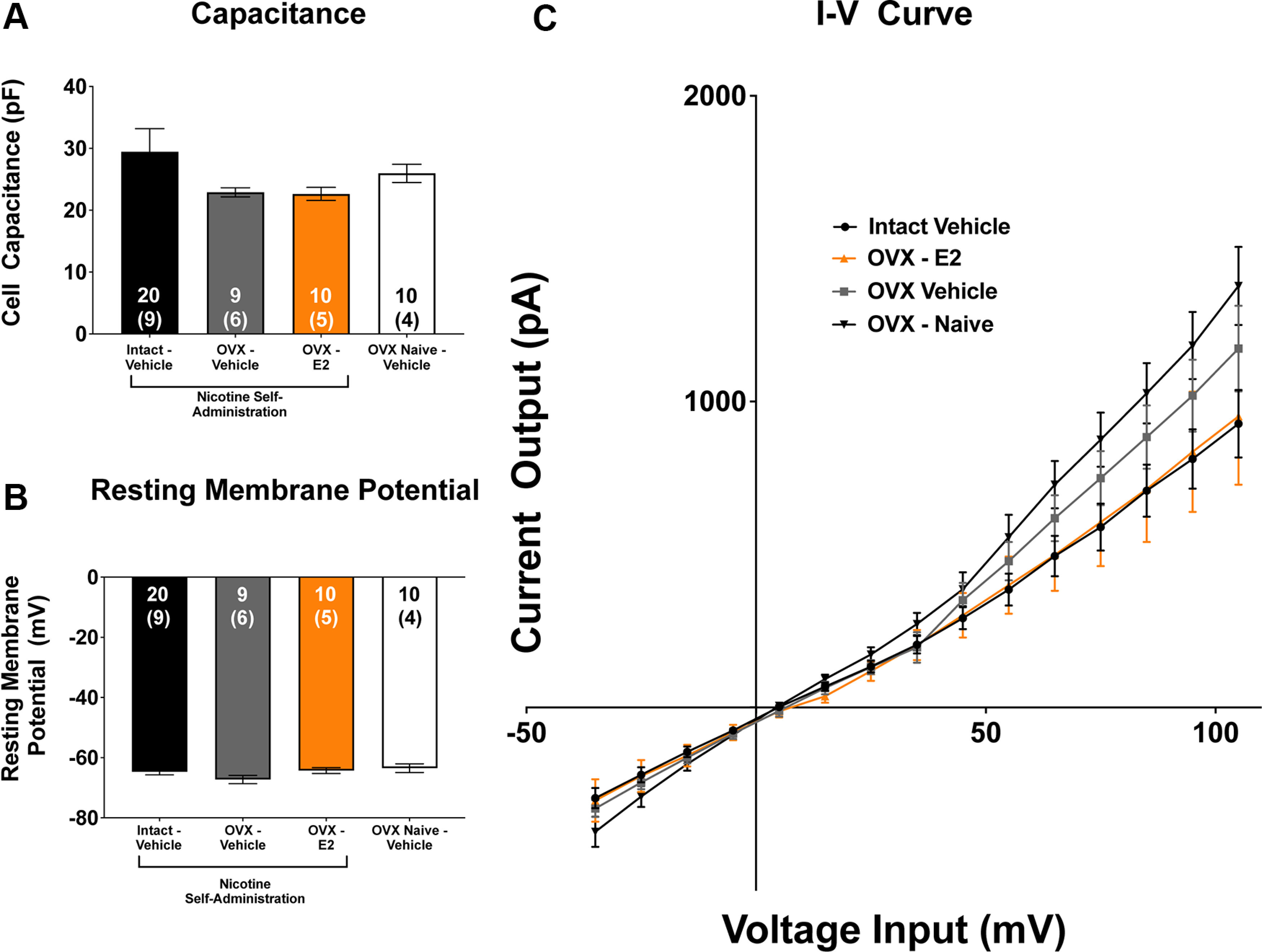
Intrinsic electrophysiological characteristics of medium spiny neurons (MSNs) do not differ as a function of ovarian hormones. (***A***) There was no main effect of group on cellular capacitance, (***B***) resting membrane potential, or (***C***) input-output responses of MSNs within the nucleus accumbens core (NAcore) (*p* > 0.05; mean ± SEM). The inset numbers represent the number of cells with the number of animals recorded from within parentheses.*Figure Contributions:* Jonna M. Leyrer-Jackson performed the experiments. Joshua S. Beckmann analyzed the data.

NAcore MSN recordings were made from acute slices derived from females in all treatment groups. LME modeling was used to assess differences in electrophysiological measurements. Analyses revealed no differences in cellular capacitance across ovary-intact-vehicle, OVX-E2, OVX-vehicle, or OVX-vehicle-nicotine naive groups (*p *>* *0.05; Fig. 7*A*). Further, no differences in resting membrane potential were observed between groups (*p *>* *0.05; Fig. 7*B*). An LME analysis of input-output curves revealed a significant effect of input (*F*_(1,17.93)_ = 143.68, *p *<* *0.0001; Fig. 7*C*). However, no effect of group or interaction was observed (*p*s > 0.05).

**Figure 8.**
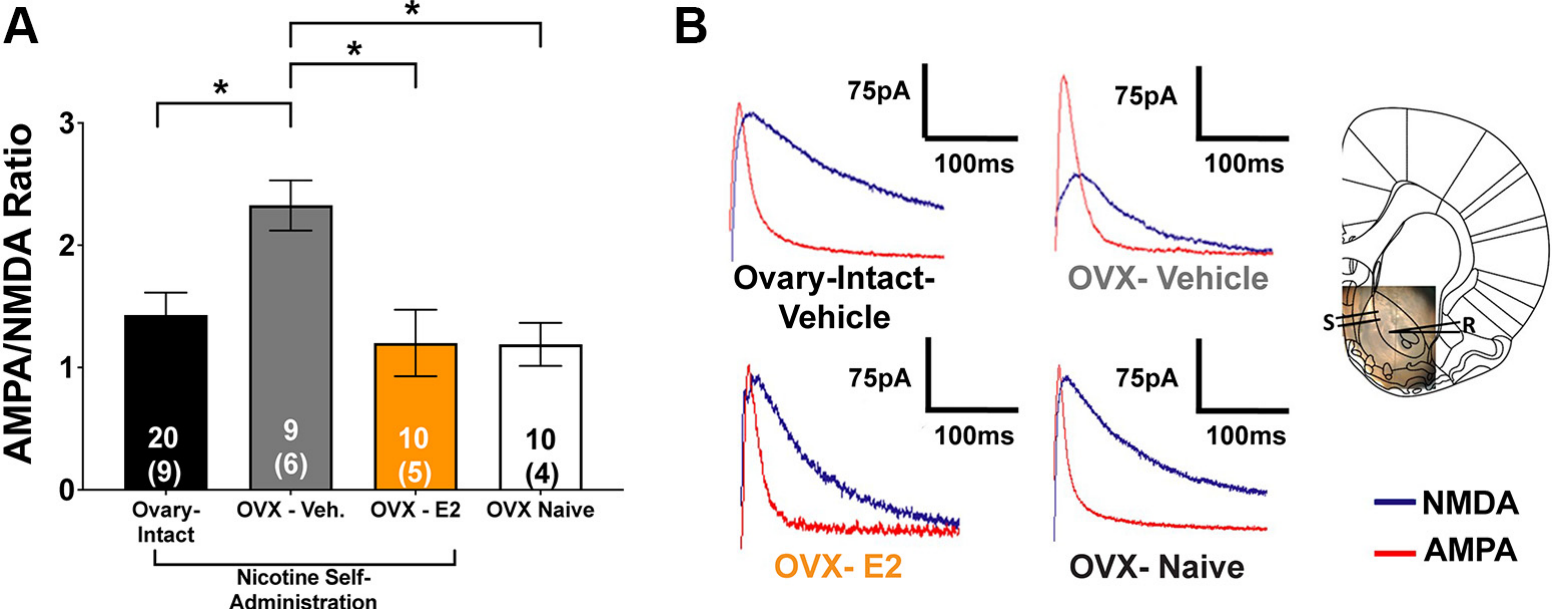
Cessation of ovarian hormones potentiates nucleus accumbens core (NAcore) medium spiny neurons (MSNs) after nicotine self-administration, which is reversed by estrogen (E2) supplementation. ***A***, A main effect of group on AMPA/NMDA ratio was found where AMPA/NMDA ratio decreased when nicotine is on board in ovary-intact females. Following ovariectomy (OVX), MSNs are potentiated in a nicotine-dependent manner. Systemic E2 supplementation (4 d) reverses this potentiation, similar to ovary-intact freely cycling females (*p <* *0.05; mean ± SEM). ***B***, Representative traces for each experimental group. In the slice representative photograph in ***B***, S represents the stimulating electrode, and R represents the recording electrode. The inset numbers represent the number of cells with the number of animals recorded from within parentheses. *Figure Contributions:* Jonna M. Leyrer-Jackson performed the experiments. Joshua S. Beckmann analyzed the data.

AMPA/NMDA ratios within the NAcore were assessed using LME modeling. A significant main effect of group was observed (*F*_(3,51)_ = 5.75, *p *<* *0.01; Fig. 7*A*), indicating that ovarian hormone manipulations altered MSN plasticity. Tukey’s *post hoc* comparisons revealed that females in the OVX-vehicle group had significantly higher AMPA/NMDA ratios than ovary-intact-vehicle, OVX-E2, and OVX-vehicle-nicotine naive groups (Fig. 8*A*).

## Discussion

The present study explored the role of ovarian hormone loss and supplementation with estrogens on nicotine self-administration, NAcore MSN synaptic plasticity, and changes in ER and nAChR mRNA within the NAcore and VTA. Here, we show that cessation of ovarian hormones reduced nicotine consumption during acquisition but did not impair lever discrimination. These results indicate that precipitous ovarian hormone loss following OVX does not impair acquisition and that conditions which impact consumption are separable from learning mechanisms. Further, our results support those by Flores et al. (2016) and our prior study (Maher et al., 2021), such that OVX decreased nicotine consumption in a volitional self-administration paradigm. Interestingly, when OVX females were given E2 supplementation with or without its metabolite, E1, reductions in nicotine consumption did not reverse regardless of the duration of hormone treatment. These results contrast with our prior study, showing that E2 partially restores nicotine consumption following OVX (Maher et al., 2021), which is discussed in more detail below. As well, E2 supplementation following acquisition did not reverse OVX-induced reductions in nicotine consumption, regardless of nicotine dose. Importantly, we show that the administration parameters of E2 alone and E1+E2 were sufficient for these hormones to alter ERα mRNA levels in the NAcore, demonstrating that the hormone treatment regimens used here allowed these estrogens to have a neurobiological impact on key brain reward regions. We further show that regardless of the hormone treatment group, nicotine self-administration suppressed estrogen-induced increases in ERα expression following OVX. Together, these studies show that ovarian hormones regulate nicotine consumption in females, and self-administered nicotine critically interacts with estrogen signaling through its receptors within the NAcore.

We also show that ovarian hormones critically regulate NAcore MSN plasticity, which was nicotine-specific. While basic electrophysiological characteristics of NAcore MSNs for each treatment group were not significantly different, abrupt loss of cycling ovarian hormones because of OVX rendered NAcore MSNs in a potentiated state following nicotine self-administration (measured via elevated AMPA/NMDA ratios), an effect that was reversed by E2 treatment. The potentiation of the NAcore MSNs was nicotine-dependent, as AMPA/NMDA ratios in OVX-vehicle nicotine-naive females were not significantly different from ovary-intact-vehicle nicotine self-administering females or from OVX-vehicle females given E2 supplementation.

### Ovarian hormones and nicotine self-administration

We found that ovary-intact females consume more nicotine than OVX females. These results are consistent with other studies demonstrating that ovary-intact females administer more nicotine during daily self-administration sessions at both low (0.015 mg/kg/infusion) and high doses (0.06 mg/kg/infusion) of nicotine (Flores et al., 2016). Interestingly, others have found that E2 supplementation with a higher dose of E2 than was used here (e.g., 25 μg up to 1 mg, as compared with 3 μg used in the current study) increases nicotine following OVX compared with vehicle-treated OVX animals (Flores et al., 2016) and promotes nicotine-induced conditioned place preference (CPP) in OVX females (Frye and Rhodes, 2006). Thus, it is possible that there is a threshold dose of E2 needed to alter nicotine behaviors. It is of note that E2 administration alone is capable of eliciting E2-induced CPP and antagonism of both ERα and ERβ within the nucleus accumbens using the nonspecific ER antagonist, ICI 182 780, inhibits E2-induced CPP (Walf et al., 2007). Thus, ERs within the accumbens appear to be involved in reward processes. While other studies have shown that E2 supplementation increases self-administration of nicotine and promotes CPP, we found that E2 supplementation in OVX females did not fully restore nicotine intake to ovary-intact levels regardless of the duration of treatment or nicotine dose used in self-administration. It is notable that only 4 d of E2 supplementation was sufficient to reverse the OVX nicotine-induced potentiation of NAcore MSNs, indicating that this reversal may precede changes in nicotine consumption because of E2 supplementation. However, it is also possible that supplementation with estrogens alone is insufficient to restore nicotine consumption to ovary-intact levels, and the presence of other circulating ovarian-derived steroid hormones are necessary to do this. Thus, future studies are needed to determine whether it is (1) the presence of various ovarian hormones, and/or (2) the cycling pattern of ovarian hormones, during the estrous cycle that is required to reach ovary-intact levels of nicotine consumption.

One critical difference between our prior study mentioned above and the current study is that OVX females in Maher et al., were given E2 supplementation for a week before initiation of nicotine self-administration (Maher et al., 2021). This may be a critically important methodological difference, given that E2 allosterically modulates nAChRs (Jin and Steinbach, 2011), and thus may impact how nicotine activates nAChRs. Thus, it is possible that E2 can partially reverse OVX-induced suppression of nicotine consumption when it is tonically given before nicotine self-administration acquisition, possibly through allosterically modulating nAChRs. This is mechanistically feasible given that E2 priming, either systemically or administered intra-amygdala, has anti-anxiety and anti-depressive like effects in OVX females (Walf and Frye, 2005), showing that E2 critically regulates nodes in the reward pathway to alter behavior.

There is evidence that estrogens impact nicotine metabolism in women. Indeed, women have higher nicotine clearance rates than men, and women taking oral contraceptives (which contain ethinyl estradiol, a synthetic estrogen that is significantly more potent than E2) show even higher nicotine metabolism rates (Benowitz et al., 2006). These could be related to ER control of the UGT glucuronosyltransferases that metabolize drugs, compounds, and hormones (Sundararaghavan et al., 2017). It should also be noted that women show greater smoking-related behaviors during the luteal phase (Carpenter et al., 2006; Terner and de Wit, 2006) when circulating estrogens and progesterone are increased following ovulation in preparation for fertilization of a mature egg. Although the effect of E1 on nicotine metabolism has not been studied, it has been shown that E1 impairs contextual fear conditioning memory (Barha et al., 2010) as well as spatial memory in female rats (Engler-Chiurazzi et al., 2012) and, thus, may lead to deleterious effects on the brain and other behaviors. Further, E1 does not protect against glutamate excitotoxicity in basal forebrain neurons (Zhao and Brinton, 2006). Thus, the anti-nicotinic actions of E2 may underlie OVX-induced reductions in nicotine consumption, whereby in an ovary-intact or E2-supplemented system, more nicotine is required to reach optimal levels because of the enhancement of nicotine metabolism by E2. In the OVX system, the absence of E2 could potentially lead to lower levels of nicotine consumption because of a lack of anti-nicotinic effects.

### Ovarian hormones, nicotine responsiveness, and ER isoform expression in the brain

The impact of nicotine on the ER isoforms, and how this affects behavior, is mostly unexplored. However, some investigations looking at nonbehavioral studies have found that nicotine reduced ERβ expression in the hippocampus and cortical regions of female rats (d’Adesky et al., 2018). Martin and colleagues showed that tobacco smoke condensate (TSC) activated estrogen signaling in human MCF-7 breast cancer cells, which was validated via competitive binding assays and transcriptional activity of the nuclear receptors (Martin et al., 2007). They also found that nicotine reduced the expression of ERs and showed that OVX Sprague Dawley female rats treated with E2 and low levels of TSC had induced levels of the ER-target gene *Pgr*, but E2 co-treated with an antagonist had reduced levels. These results suggest that TSC may have an estrogenic effect, supported by another study by Niwa and colleagues in MCF-7 breast cancer cells, which showed that TSC induced kinase pathways that activate ERα (Niwa et al., 2017). Others have shown that E2 treatment in rainbow trout induced ERα mRNA expression but suppressed ERβ levels in the liver (Casanova-Nakayama et al., 2018). The effects of TSC may be through specific nicotine receptor subunits (*Chrna7* and *Chrnb2*), which induce signaling cascades that control physiological responses. Whether the effects of nicotine on estrogen signaling occur in the brain to regulate behavior is unknown, but here we indicate that nicotine suppresses the effects of E2 on the expression of ERα in the NAcore and VTA regions.

Hinfray and colleagues used zebrafish and mixture concentration-response (MCR) modeling to show that genistein, a phytoestrogen, and E2 treatments increased ERα and ERβ activity in human glial U251-MG cells. In addition, they showed that E2 and genistein treatments in *Cyp19a1b*-GFP transgenic zebrafish activated radial glial cells and *Cyp19a1b* in the brain (Hinfray et al., 2018). The *Cyp19a1b* gene is commonly referred to as aromatase B or brain aromatase (Mouriec et al., 2009), which has estrogen response elements (EREs) in its promoter where ERα and ERβ can bind and control expression (Menuet et al., 2005; Le Page et al., 2006, 2008). In addition, studies in Indian major carp showed that a 6-d E2 treatment significantly increased *Cyp19a1b* expression in the brain, which was suppressed by aromatase inhibitors (Gupta et al., 2017). The effects of estrogen on the *Cyp19a1b* gene might be cell-specific and have only been reported in radial glial cells or astrocytes (Menuet et al., 2005; Le Page et al., 2006, 2008). Using the *Cyp19a1b*-GFP transgenic zebrafish, Serra et al., demonstrated that triclosan is an inhibitor of estrogen signaling mechanisms (Serra et al., 2018). While these studies are diverse and do not explicitly study behavior, they provide a mechanistic understanding of how the ER isoforms may be affected and how substances such as triclosan can suppress these events. Future studies using this model with nicotine exposure will help reveal whether nicotine directly targets ER isoform transcriptional activity or if nicotine regulates neuronal plasticity via suppression of the ER isoform mRNAs.

Our study is the first to show that nicotine regulates ERα at the transcriptional level in the NAcore and VTA of females. Here, we show that in an OVX system with E2 present, there are increases in VTA α7 nAChR and ERα mRNA, both of which are suppressed by nicotine. Interestingly, increases in α7 nAChR activation block nicotine-induced dopamine release from dopaminergic neurons in the VTA (Melis et al., 2013). The functional consequence of the mRNA results presented in the current study may be an enhancement of VTA dopamine firing activity, which may modulate NAcore MSNs (Pontieri et al., 1996). Importantly, nicotine increases dopamine release in the NAcore (Di Chiara and Imperato, 1988), whereby dopamine then activates dopamine (D)1 or D2 receptors (Gerfen et al., 1990; Gerfen and Surmeier, 2011) located on MSNs (Hedreen, 1981; Bolam, 1984; Chang and Kitai, 1985; Meredith, 1999). Given that the VTA sends strong modulatory dopaminergic input onto NAcore MSNs, future studies are needed to determine whether these transcriptional changes in the VTA result in altered dopaminergic modulation of MSN plasticity in the NAcore, and whether this circuit-level mechanism underlies nicotine self-administration in females.

### Altered plasticity of NAcore MSNs following OVX in nicotine self-administering females

Our prior studies have examined plasticity during reinstatement of nicotine seeking in male rats, which is a protracted time point from nicotine self-administration (Gipson et al., 2013b; Namba et al., 2020; Leyrer-Jackson et al., 2021). In our first paper examining NAcore plasticity following nicotine self-administration, we found that initiated nicotine seeking in a cue-induced reinstatement model potentiated NAcore MSNs, which then collapsed within 45 min (similar to when active lever pressing declined; Gipson et al., 2013a). These results are similar to the rapid, transient plasticity found after 15 min of cue-induced cocaine-seeking (Gipson et al., 2013a). Here, we examined glutamatergic plasticity in NAcore MSNs immediately following nicotine self-administration in females, which has not yet been established. Further, the current study is the first to characterize steroid hormone-induced regulation of NAcore glutamate plasticity in nicotine-experienced females, demonstrating that MSN neurophysiology following nicotine self-administration is critically regulated by neuroendocrine mechanisms. To our knowledge, the only other study examining MSN plasticity with a self-administered drug on board showed a collapse of structural potentiation (measured via dendritic spine morphology) following the reintroduction of intravenous cocaine during an initiated reinstatement session in males (Spencer et al., 2017). Although not using a nicotine self-administration paradigm, one recent study showed that NAcore MSN excitability was decreased with E2 replacement following OVX, consistent with our current findings (Proaño and Meitzen, 2020). Together, the findings presented here and those of others demonstrate that that MSN physiology within the NAcore of females is critically regulated by the neuroendocrine system.

In concurrence with others, we show that ERs (including ERα and ERβ) are densely expressed within the NAcore of the striatum (Satta et al., 2018; Tonn Eisinger et al., 2018; Yoest et al., 2019). As well, we found localization of ERs on NAcore neurons as well as VTA dopaminergic cell bodies. Within the striatum, ERα is coupled to both groups I and II mGluRs, which activate G_q_ or G_i/o_, respectively, to activate and inhibit the cellular transcription factor, cAMP response element-binding protein (CREB) phosphorylation (Grove-Strawser et al., 2010). ERβ, however, is only coupled to Group II mGluRs and thus can only inhibit CREB phosphorylation (Grove-Strawser et al., 2010). Given that E2 has been linked to changes in dendritic plasticity through its interactions with the metabotropic glutamate receptor five subtypes mGluR5; (Peterson et al., 2015), a receptor commonly associated with addiction (Kenny and Markou, 2004; Jones and Bonci, 2005; Volkow et al., 2019) and is involved in drug-seeking behavior (Wang et al., 2013), and ERs form complexes with mGluRs (Grove-Strawser et al., 2010), it is possible that ERs can influence nicotine seeking and MSN physiology in females. This possibility is also supported by evidence showing that E2-mediated activation of mGluR5 induces elevations of phosphorylated CREB in MSNs of female rats (Grove-Strawser et al., 2010). Furthermore, E2 directly regulates dopamine release within the striatum, giving rise to physiological changes in reward pathways (Becker, 1990; Mermelstein et al., 1996; Xiao and Becker, 1998). Interestingly, nicotine has been shown to decrease levels of biologically active E2 (Mueck and Seeger, 2005) and thus could reduce dopaminergic tone within the striatum. Given these findings, E2 is capable of regulating both dopaminergic tone through its interactions with ERs located on dopaminergic terminals, as well as MSN excitability through ER-mGluR complexes. In the current study, we show that OVX vehicle-treated rats show elevated AMPA/NMDA ratios, an effect that is reversed via systemic E2 administration in OVX animals. Thus, it is possible that nicotine consumption following the cessation of hormones promotes a metaplastic state of MSNs, rendering them more responsive to decreased dopaminergic tone. Further, we show that nicotine suppresses estrogen-induced increases in ERα transcription within the NAcore following OVX. Future studies are needed to determine whether this is a neurobiological alteration that functionally impacts NAcore glutamatergic plasticity.

It is important to discuss the disparities in the results between neurophysiology and behavior in the current studies. As discussed above, 4 d of E2 supplementation following nicotine self-administration acquisition reversed OVX-induced increases in AMPA/NMDA ratio in NAcore MSNs. However, neither duration of E2 treatment nor the addition of E2’s metabolite, E1, reversed the OVX-induced decreases in nicotine consumption. It is important to note that the brain and the environment interact bidirectionally, ultimately contributing to the emission of behaviors (Strickland et al., 2022). As such, neurobiological mechanisms may be rapidly impacted by stimuli; however, behavior is complex and likely requires various other factors, including the presence of other hormones that regulate the female reproductive cycle (e.g., progesterone, luteinizing hormone, and follicle-stimulating hormone), restoration of the hypothalamic-pituitary-ovarian axis (Mikhael et al., 2019) among others. Further, rats in both experiments received daily tonic injections of the same doses of estrogens, which does not mimic the cycling ovarian hormone milieu during the estrous cycle. E2 rapidly increases ER expression (Ihionkhan et al., 2002), as well as ER transcriptional activity, which happens within minutes, as previously shown by chromatin immunoprecipitation of ER docking at EREs on gene promoters (Vega et al., 2006) and microarray data for timepoints 1–24 h (Lin et al., 2004), which can have lasting effects (Lin et al., 2004). Thus, 4 d of tonic E2 may have been sufficient to reverse OVX-induced increases in AMPA/NMDA ratio but was insufficient to restore nicotine consumption back to ovary-intact levels.

### Limitations of the current study

One limitation of the current study is that a yoked saline control group was not included. One previously published paper, however found no differences in IPSCs recorded from the ventral pallidum of naive versus yoked saline male rats and pooled these data for analysis (Kupchik et al., 2014). Additionally, animals receiving noncontingent saline did not differ from injection-naive animals in AMPA/NMDA ratios in accumbens shell MSNs (Kourrich et al., 2007). Although there are differences relative to the current study, this previous study indicates that it is likely that there are no differences in the NAcore AMPA/NMDA ratio of naive versus yoked saline rats. Additionally, the electrophysiological experiments in the current study were conducted without GABA_A_ receptor antagonists within the bath, thus leaving an intact network of both GABAergic and glutamatergic activity. Therefore, it is possible that intact inhibitory currents could alter the AMPA/NMDA currents presented in the current study. However, we did not observe polysynaptic currents within the whole EPSC or NMDA-mediated EPSCs, suggesting that intact GABAergic activity would not significantly alter the results reported here. However, future studies inhibiting GABAergic activity would help to isolate glutamatergic activity onto MSNs and may lend additional insight into subcellular mechanisms involved in our results reported here.

It should also be noted that in some control animals (specifically for electrophysiological and RT-qPCR endpoints), no sham surgeries were conducted. This is a limitation of the current set of studies, as the stress from surgery could impact outcomes. However, given that no differences in electrophysiological measures were observed between intact-sham groups and intact groups without shams conducted, it is likely that the lack of sham surgery had little impact on the variables measured. Notably, all control animals that underwent nicotine self-administration received sham surgery, and thus this is not a limitation of the behavioral outcomes.

In conclusion, here, we show that circulating ovarian hormones play significant roles in nicotine self-administration, and in the presence of self-administered nicotine, E2 can alter MSN synaptic physiology within the NAcore. However, the current set of studies shows that estrogen replacement following OVX likely needs to occur before nicotine self-administration acquisition for estrogens to increase consumption. However, we show that NAcore MSNs from OVX females are critically regulated by 4 d of exogenous E2 treatment and that this neurobiological effect dissociates from nicotine consumption. Together, these studies suggest that although neural function may be rapidly impacted by estrogen replacement, the impacts on behavior are much more complex. Future studies should determine whether the reversal of OVX-induced reductions in nicotine consumption requires the presence of additional steroid hormones (such as progesterone). To our knowledge, these are the first studies to explore the role of E2 on nicotine self-administration and associated accumbens plasticity and the first to include E1 in these effects. Our results also demonstrate that cessation of ovarian hormones may induce a form of metaplasticity, similar to that observed in males following cocaine withdrawal (Moussawi et al., 2009) in NAcore synapses, which is reversed by E2 treatment. Taken together, these studies highlight important contributions of E2 to nicotine use and underlying NAcore glutamatergic plasticity. Future preclinical studies are needed to further decipher the contributions of other endogenously cycling hormones and exogenously administered natural and synthetic hormones to nicotine use motivation and the underlying neurobiology. Future studies using ER isoform-specific knock-out animals to determine the function of each isoform in regulating accumbens plasticity and the impact of nicotine would prove beneficial. Indeed, systematic assessments of ovarian hormone-nicotine relationships are critical to yield translational impacts that will improve treatment strategies to promote smoking cessation in women. The importance of novel discovery in this domain is underscored by the sex differences in vulnerability to TUD, that women have greater challenges than men regarding abstinence from smoking, and that nicotine craving and propensity for relapse are impacted by the phase of the menstrual cycle and estrogen levels.

## Synthesis

Reviewing Editor: Christie Fowler, University of California, Irvine Decisions are customarily a result of the Reviewing Editor and the peer reviewers coming together and discussing their recommendations until a consensus is reached. When revisions are invited, a fact-based synthesis statement explaining their decision and outlining what is needed to prepare a revision will be listed below. The following reviewer(s) agreed to reveal their identity: Stephanie Caligiuri, Laura O’Dell.

This manuscript has been reviewed by two experts in the field. Both reviewers appreciated the responsiveness of the authors to the prior review comments. It was noted that the addition of the E1 and E2 supplementation and additional time points has strengthened the findings presented in the manuscript. However, there was some concern in the interpretation of these data relative to the behavioral effects, when attempts are made to compare findings from OVX rats to intact females.

Specifically, it should be recognized that the discrepancy in behavioral versus neurochemical effects may be related to different thresholds in altering behavior versus brain changes. Given prior work, it seems that higher doses of estradiol are needed to effectively normalized nicotine IVSA in OVX rats following systemic injections of this hormone. The authors characterize the prior work as using “supraphysiological levels” to alter nicotine IVSA. However, the existing published nicotine IVSA data in combination with the present data in this report suggest that higher doses of estradiol are needed to normalize nicotine IVSA in OVX rats to intact control levels. Arguing that the doses in prior work are not in a physiological range fails to recognize that a particular dose range may be required to normalize nicotine IVSA in OVX rats. The present wording will likely cause future readers to disregard prior work, and this is inappropriate in the absence of information that has determined the precise brain levels of this hormone with such systemic administration. Importantly, the possibility exists that the threshold for altering behavioral effects versus neurochemical effects falls within different dose ranges, or alternatively, pulsatile patterns of hormones across time in the intact state may be important. Therefore, the findings related to prior work should be discussed in a more balanced manner, or studies need to demonstrate the differences in estrogen levels within target brain regions after systemic injections, compared to the intact female.
